# A “Migrant Friendly Hospital” Initiative in Geneva, Switzerland: Evaluation of the Effects on Staff Knowledge and Practices

**DOI:** 10.1371/journal.pone.0106758

**Published:** 2014-09-08

**Authors:** Patricia Hudelson, Melissa Dominice Dao, Thomas Perneger, Sophie Durieux-Paillard

**Affiliations:** 1 Department of Community Medicine, Primary Care and Emergency Medicine, Geneva University Hospitals, Geneva, Switzerland; 2 Primary Care Service, Department of Community Medicine, Primary Care and Emergency Medicine, Geneva University Hospitals, Geneva, Switzerland; 3 Division of Clinical Epidemiology, Geneva University Hospitals, Geneva, Switzerland; 4 Programme Santé Migrants, Department of Community Medicine, Primary Care and Emergency Medicine, Geneva University Hospitals, Geneva, Switzerland; Public Health Agency of Barcelona, Spain

## Abstract

**Background:**

International migration poses important challenges to European health care systems. The development of “migrant friendly hospitals” has been identified as a priority in both Europe and Switzerland.

**Methods:**

A multi-pronged initiative was developed at Geneva University Hospitals (HUG) to improve staff knowledge and use of existing “migrant friendly” resources. A self-administered questionnaire was sent pre and post-intervention to random samples of 4 major professional groups with direct patient contact at the HUG. The questionnaire assessed staff knowledge, attitudes and reported practices regarding the care of migrant patients.

**Results:**

Overall response rate was 51% (N = 1460) in 2010 but only 19% (N = 761) in 2013 owing to an institutionally imposed change in survey method. Despite these difficulties, and after adjusting for sample differences, we found that respondents in 2013 were significantly more likely to have received training in how to organize an appointment with an interpreter, how to work with an interpreter and about health and social services available for migrant patients. Respondents were also significantly more likely to have used several Migrant Friendly structures at the HUG. Use of, preference for and perceived skill at working with professional interpreters all improved, and respondents were both more likely to be encouraged by their supervisors to use professional interpreters, and less likely to be encouraged to look for alternative solutions for communicating with non francophone patients. Finally, 2013 respondents encountered fewer difficulties caring for migrant patients, although lack of time and language barriers continued to be the most important sources of difficulty.

**Conclusion:**

Our results suggest that an institution-wide information campaign may contribute to increased awareness and use of migrant friendly resources by clinical staff. Hospital commitment and financing, along with inter-departmental participation in all activities were important in creating and maintaining project visibility, and in contributing to a migrant friendly institutional culture.

## Background

### The need for Migrant Friendly hospitals

International migration poses important challenges to European health care systems, who struggle to meet the needs of increasingly diverse patient populations [1], [2], [3], [4]. Most European countries lack detailed information on the health of migrants [5], [6], [7], and the data that do exist reflect conceptual and methodological difficulties related to the social and legal heterogeneity of the migrant population. There is no consensus on a single definition of ‘migrant’ [8]; the UN defines a long term migrant as “a person who moves to a country other than that of his or her usual residence for a period of at least a year” [9], a definition which fails to reflect the vast diversity among migrants with regards to the reasons for and processes of migration, as well as their living conditions and legal rights in the host country. Nonetheless, a number of studies suggest that migrants as a whole are more vulnerable to certain chronic and communicable diseases, mental health problems, occupational health hazards and injuries [10]. Many migrants are exposed to a range of health risks before, during and after migration, including exposure to disease, poor living conditions, precarious living and work conditions, and psychological stresses associated with the process of migration. In addition, migrants often encounter social, cultural, linguistic, legal and economic barriers to health care in their host countries [6], including direct and indirect discrimination on the part of health services [11].

To ensure equity in health and health care for all patients, health systems need to consciously and systematically incorporate the needs of migrants into all aspects of health service planning and implementation [12]. Towards this end, initiatives have been developed in the USA and Europe aimed at building “culturally competent” [13] or “migrant friendly” [14], [15] health care institutions. These initiatives emphasize the importance of facilitating access to professional interpreter services, routine collection of patient language data, training health-workers in cross cultural communication, and adapting information to migrants' health literacy levels [16]. However, developing clinical and administrative structures adapted to migrant patient needs is not enough. Staff must be made aware of these services, understand when and how to make use of them, and be convinced of their usefulness. An institutional culture consisting of shared values, norms and practices [17] around the care of migrant patients must be developed for migrant friendly hospitals to be effective.

### Building a “migrant friendly” institutional culture at the University Hospitals of Geneva

As part of a “National Migration and Public Health Strategy” [18], [19], the Federal Office of Public Health (FOPH) has promoted the development of “Migrant Friendly Hospitals” in Switzerland [20]. The University Hospitals of Geneva (HUG) is one of five hospital groups funded under this initiative. The HUG is an 1800-bed hospital group serving a diverse population. Forty percent of Geneva residents are of foreign nationality (190 nationalities) [38] and 25% of Geneva residents speak a language other than French as their primary language [39]. At the HUG, 50% percent of patients are of foreign nationality [40], [41], and one in twelve patients speaks no French at all [42].

For over 15 years, the HUG has been developing “migrant friendly” services in an attempt to ensure quality care for all patients regardless of their language, culture or socioeconomic status. These include several specific primary care clinics for asylum seekers, uninsured patients and migrant children [21], a pediatric ethnopsychiatry consultation, a consultation for victims of war and torture [22], a community interpreter service run by the Geneva Red Cross [23] and a cultural consultation service to aid clinicians who encounter cultural barriers with their patients [24]. However, there was no systematic and widespread provision of information to staff about these services and how to use them. Previous research [25], [26], [27] and anecdotal information indicate that clinicians often feel unprepared to deal effectively with the needs of such a diverse patient population, and were unaware of the resources available to them. In order to address these issues and strengthen “migrant friendly” institutional culture, we developed a multipronged program at the hospital.

### MFH Programme at the HUG

Because our aim was to create a base of shared knowledge and practices around migrant care, we began by creating an interdepartmental and interprofessional working group (“Health for All Network”) with representation from the main clinical departments, and which was responsible for developing and implementing the project. This group identified the following priority activities, which were implemented over a 3 year period (2010–2013):

- Creation of a reference-nurse post at the hospital for migrant care issues. This individual provides information and support to staff across all hospital departments regarding care of migrant patients.
- Inclusion of patient language data in the electronic patient file, in order to facilitate timely identification of patients requiring interpreter services [28].
- Promotion of a national telephone interpreting service in 4 emergency services at the HUG, where access to face-to-face interpreters is rare due to time constraints and scheduling difficulties.
- Brief presentation to all new staff during an obligatory staff orientation day about interpreter services and other “migrant friendly” services at the HUG.
- Development and dissemination of brochures containing information about the “Health For All Network” [29]; migrant friendly services at the HUG [30]; and when and how to work with an interpreter [31].
- Organization of a number of public events to bring attention to the Health For All Network and its activities, including a mid-day “happening” at the hospital involving music, free soup, a public debate about racism in health care [32], and distribution of brochures; creation a brief video about the Network, shown on local television and the HUG website [33]; an article in the internal HUG newsletter sent to all staff, and organization of a movie series on cultural diversity at a local cinema, with subsidized entree fees for HUG staff.

The aim of the present study was to evaluate the impact of the program on staff knowledge, attitudes and practices regarding the care of migrant patients.

## Methods

### Evaluation design, sample, and data collection

In order to evaluate the impact of our actions on staff knowledge, attitudes and practices we conducted a staff survey before the project began (fall 2010) and again in April/May 2013 (See additional file: Questionnaire S1).

On both occasions, a self-administered questionnaire was sent to random samples of 4 major professional groups with direct patient contact, across 11 medical departments at the HUG. The four groups were doctors, nurses, nurse aides, and “other health professional”, comprised of other clinical staff (such as dieticians, psychologists, physiotherapists, etc.) and social workers.

The 2010 survey was sent to the home addresses of selected staff. After the initial mailing, 2 reminders were sent at 3 week intervals. In 2013, due to a new institutional paper-saving policy, we were obliged to use an on-line questionnaire which was sent to participants' HUG email addresses. Two email reminders were sent at 2 week intervals.

In 2010, based on previous survey experience at the HUG, we anticipated a response rate around 50%. Sample sizes of 750 doctors, 753 nurses, 750 nurses' aides and 714 “others” were selected. Sample size was determined to have sufficient power (90%) and low type 1 error probability (5%) to detect differences between professional groups of 0.25 standard deviation on any continuous variable.

We had no previous experience to indicate what sort of response rate to expect with an email based questionnaire. Some staff do not use computers during the workday (nurse aides, for example), or may not access their professional email at home. We thought that doctors might be swamped with emails or too busy with clinical work to respond to an internet-based questionnaire. We anticipated a lower response rate for the 2013 survey, and increased our sample size accordingly, to include all 1160 eligible doctors, an equal number of nurses, all 1103 eligible nurse aides, and all 581 “other” (social workers and other clinical staff).

### Ethics statement

As a quality assessment project that entails minimal risk to participants, this study was exempted from review by the Geneva University Hospitals Research Ethics Commission. In the cover letter or email accompanying the questionnaires, we explained that participants were randomly selected, that results would be analyzed anonymously, and that participation was voluntary. Consent was considered given if the respondent completed and returned the questionnaire.

### Study variables

The self-administered questionnaire explored respondents' knowledge, attitudes and practices related to the care of immigrant patients. Most items were newly developed or adapted by us. The questionnaire was written in French. The questionnaire was pretested and finalized with members of the Health for All network.

Questionnaire items/sections analyzed in this article include sociodemographic and professional characteristics (6 questions); sources of difficulties encountered when caring for migrant patients (13 Likert-type scales, from “rare cause of difficulty” to “very frequent cause of difficulty”); training received on topics related to care of migrant patients (7 yes/no questions); use of migrant friendly services in the last 6 months (8 yes/no questions); workplace encouragement to use professional interpreters (1 multiple-choice question, one response possible); use of different types of interpreters in the last 6 months (6 questions, from “never” to “more than 20 times”); preferred type of interpreter (1 multiple-choice, one response possible); and self-assessment of patient care skills (9 Likert-type scales, from “not at all competent” to “very competent”). The exact wording of items appears in the results tables.

### Analysis

In 2013 there were almost no respondents from the Department of Genetics and Laboratories, and the Department of Imaging. In the latter case, it appears that radiology technicians may have been excluded from the sample by mistake. For these reasons, we excluded from our analyses all administrative staff and all respondents from the Department of Genetics and Laboratory and the Department of Medical Imaging.

We report the raw comparisons (without adjustment) and also adjusted by professional category, hospital department, function (senior doctor or nurse, vs. staff nurse or resident), and Swiss versus other citizenship.

Multivariate analyses were performed using logistic regression. For ordinal variables, such as the skill ratings scored between 1 and 5, response options 4–5 are compared with options 1–3. For nominal variables (such as the service attitude question), we created a binary variable for each response option, which is then compared to all other options.

In all cases the result is an odds ratio of giving a more positive response in 2013 compared with 2010. The analyses were performed using SPSS version 18 software.

## Results

Overall response rate was 51% (N = 1460) in 2010 but only 19% (N = 761) in 2013. Response rate was lower for all 4 professional categories in 2013, but especially so for nurse aides (from 42.9% in 2010 to 6.3% in 2013).

Sample characteristics were also considerably different between the two surveys with regards to four variables: profession, staff position, department and nationality (Table 1). The 2013 sample had proportionally more Swiss respondents, more senior staff respondents, more doctors, many fewer nurse aides, and more respondents from the Departments of Community medicine, Psychiatry, Obstetrics and Gynecology, Child and Adolescent Health, and Surgery.

**Table 1:** Respondent characteristics (after correction).

	2010	2013	P value
	N (%)	N (%)	
**N**	1336	745	
**Sex:**			0.11
Male	364 (27.3)	220 (30.7)	
Female	967 (72.7)	496 (69.3)	
**Number of years at the HUG:**			0.57
<1 year	85 (6.4)	57 (7.7)	
1–5 years	307 (23.0)	194 (26.1)	
6–10 years	346 (25.9)	151 (20.4)	
11–20 years	340 (25.4)	185 (24.9)	
Plus de 20 years	258 (19.3)	155 (20.9)	
**Nationality:**			0.003
Swiss	676 (51.1)	431 (58.0)	
Other	646 (48.9)	312 (42.0)	
**Senior staff position:**			<0.001
Yes	189 (14.1)	174 (23.4)	
No	1147 (85.9)	571 (76.6)	
**Profession:**			<0.001
Social worker/public health nurse	79 (5.9)	55 (7.4)	
Nurse	413 (30.9)	230 (30.9)	
Nurses' aide	308 (23.1)	69 (9.3)	
Doctor	300 (22.5)	243 (32.6)	
Other	236 (17.7)	148 (19.9)	
**Department:**			<0.001
Anesthesiology, Pharmacology and Intensive Care (APSI)	220 (16.5)	61 (8.2)	
Surgery	92 (6.9)	70 (9.4)	
Child and Adolescent	134 (10.0)	101 (13.6)	
Gynecology and Obstetrics	44 (3.3)	42 (5.6)	
Community Medicine, Primary Care and Emergency Medicine (DMCPRU)	96 (7.2)	82 (11.0)	
Internal Medicine, Rehabilitation and Geriatrics/Specialty Medicine	419 (31.4)	166 (22.3)	
Neurosciences	95 (7.1)	50 (6.7)	
Psychiatry	236 (17.7)	173 (23.2)	

Between 2010 and 2013, there were several encouraging changes in respondents' knowledge, attitudes and practices regarding care of migrant patients at the HUG. First, we have been fairly successful in reaching staff: nearly half of respondents (46.2%, n = 343) had heard of the HUG Health for All Network in 2013. In addition, respondents in 2013 were more likely to have received training in how to organize an appointment with an interpreter (adjusted OR 1.6 [1.2–2.0], p<.001), how to work with an interpreter (adjusted OR 1.4 [1.1–1.8], p = 0.013), and about health and social services available for migrant patients (adjusted OR 1.6 [1.2–2.1], p<0.001). No change was observed with regards to training on religious and cultural characteristics of specific immigrant populations, but no new training activities on these topics were offered during the project period.

In 2013, respondents were also more likely to have had contact with several Migrant Friendly structures at the HUG. These included the primary care clinic for asylum seekers (adjusted OR 1.6 [1.3–2.0], p<0.001), the Red Cross interpreter services (adjusted OR 1.9 [1.5–2.4], p<0.001), and the Cultural Consultation support service for clinicians (adjusted OR 1.9 [1.4–2.6], p<.0.001). Even though no specific information was disseminated about the consultation for victims of war and torture, respondents in 2013 were also significantly more likely to have had contact with this structure (adjusted OR 1.9 [1.4–2.5], p<0.001). Finally, respondents in 2013 were more likely to be encouraged by their supervisors to use professional interpreters (adjusted OR 1.7 [1.3–2.1], p<0.001), and less likely to be encouraged to look for alternative solutions for communicating with non francophone patients (adjusted OR 0.7 [0.5–0.9], p = 0.002).

Use of, preference for and perceived skill at working with professional interpreters all improved (Table 2). 2013 respondents were significantly more likely to have used a professional interpreter, either face-to-face or over-the-phone in the last 6 months, and significantly less likely to have used a bilingual staff member to translate. However, use of patients' family members or friends to translate remained stable. 2013 respondents were also more likely to prefer working with professional interpreters and less likely to prefer working with patients' family or friends. 2013 respondents rated their skill at working with a professional interpreter more highly than respondents in 2010, although self-assessments of other patient care skills remained stable (Table 3).

**Table 2:** Respondents' use of and preference for different types of interpreters in 2013, compared to 2010.

Respondent has used the following at least once in the last 6 months:	2010	2013	Unadjusted analysis		Adjusted analysis*	
	N (%)	N (%)	OR (95% CI)	P value	OR (95% CI)	P value
Patient's family member or friend	1172 (88.7)	623 (84.3)	0.7 (0.5–0.9)	0.005	0.8 (0.6–1.0)	0.092
Patient's child (under 18 years of age)	449 (34.7)	257 (35.3)	1.0 (0.8–1.2)	0.81	0.9 (0.8–1.1)	0.49
Myself (I speak a language other than French)	997 (75.9)	586 (80.1)	1.3 (1.0–1.6)	0.033	1.2 (1.0–1.6)	0.081
Bilingual staff member	1076 (81.5)	541 (74.4)	0.7 (0.5–0.8)	<0.001	0.7 (0.6–0.9)	0.009
Red Cross interpreter, face-to-face	526 (39.7)	417 (65.7)	2.0 (1.7–2.4)	<0.001	1.4 (1.2–1.8)	0.001
Red Cross interpreter, over-the-phone	160 (12.1)	194 (26.5)	2.6 (2.1–3.3)	<0.001	2.1 (1.7–2.7)	<0.001
**If given the choice, respondent prefers:**						
Patient's family member or friend	366 (27.6)	126 (17.0)	0.5 (0.4–0.7)	<0.001	0.7 (0.5–0.9)	0.002
Red Cross interpreter	464 (35.0)	398 (53.6)	2.1 (1.8–2.6)	<0.001	1.6 (1.3–2.0)	<0.001
Bilingual staff member	388 (39.2)	168 (22.6)	0.7 (0.6–0.9)	0.001	0.8 (0.7–1.0)	0.10
Don't know	109 (8.2)	51 (6.9)	0.8 (0.6–1.2)	0.27	0.9 (0.6–1.2)	0.43

*Adjusted by professional category, hospital department, function (senior doctor or nurse, vs. staff nurse or resident), and Swiss versus other citizenship.

**Table 3:** Proportions of respondents who highly rated their skills in caring for migrant patients (at 4 or 5 on a scale from 1 to 5) in 2013 vs. 2010.

	2010	2013	Unadjusted analysis		Adjusted analysis*	
Patient care skill	N (%)	N (%)	OR (95% CI)	P value	OR (95% CI)	P value
Take the patient's social history	449 (35.2)	308 (41.9)	1.3 (1.1–1.6)	0.003	1.0 (0.8–1.2)	0.85
Identify the patient's literacy level in French	507 (39.5)	329 (44.9)	1.3 (1.0–1.5)	0.017	1.2 (1.0–1.4)	0.12
Negotiate a treatment plan with the patient and his family	402 (31.9)	277 (38.0)	1.3 (1.1–1.6)	0.005	1.1 (0.9–1.3)	0.58
Evaluate the patient's understanding of his/her health problem	404 (31.7)	244 (33.5)	1.1 (0.9–1.3)	0.41	0.9 (0.7–1.7)	0.40
Discuss the risks and advantages of complementary and traditional medicine treatments used by the patient	188 (14.9)	128 (17.4)	1.2 (0.9–1.5)	0.13	1.0 (0.8–1.3)	0.76
Identify any cultural practices of the patient that may have an impact on his/her care	295 (23.1)	168 (22.9)	1.0 (0.8–1.2)	0.93	1.0 (0.8–1.3)	0.99
Work effectively with a professional interpreter	616 (48.5)	445 (61.0)	1.7 (1.4–2.0)	<0.001	1.4 (1.1–1.7)	0.001
Explore possible trauma experienced by the migrant patient	254 (20.0)	181 (24.7)	1.3 (1.1–1.6)	0.013	1.1 (0.9–1.5)	0.23
Refer the migrant patient to appropriate social and medical services	306 (24.0)	227 (31.2)	1.4 (1.2–1.8)	<0.001	1.1 (0.9–1.4)	0.28

*Adjusted by professional category, hospital department, function (senior doctor or nurse, vs. staff nurse or resident), and Swiss versus other citizenship.

Finally, we also observed some important changes with regards to respondents' experiences of difficulty in caring for migrant patients (Table 4). In 2013, respondents were less likely to rate lack of experience with migrant patients, patient's lack of French, lack of access to professional interpreters, and lack of translated patient materials as important sources of difficulty. However, it is worth noting that lack of time and the patient's lack of French were the most important sources of difficulty in both 2010 and 2013 (over half of respondents gave these an importance rating of 4 or 5 on a scale of 1–5).

**Table 4:** Important sources of difficulty working with migrant patients in 2013 as compared to 2010 (at 4 or 5 on a scale from 1 to 5).

	2010	2013	Unadjusted analysis		Adjusted analysis*	
Source of difficulty	N (%) à 4–5	N (%) à 4–5	OR (95% CI)	Valeur p	OR (95% CI)	Valeur p
Patient's lack of French	815 (61.9)	403 (55.7)	0.78 (0.64–0.93)	0.007	0.81 (0.67–0.98)	0.029
Patient's lack of knowledge of how hospital functions	564 (43.4)	242 (33.2)	0.65 (0.54–0.79)	<0.001	0.67 (0.55–0.82)	<0.001
Lack of experience with migrant patients	293 (22.4)	119 (16.1)	0.67 (0.53–0.84)	0.001	0.70 (0.55–0.89)	0.004
Lack of access to professional interpreters	388 (29.8)	142 (19.5)	0.57 (0.46–0.71)	<0.001	0.70 (0.55–0.88)	0.002
Lack of written patient information in patients' languages	671 (51.9)	281 (38.8)	0.59 (0.49–0.71)	<0.001	0.68 (0.56–0.83)	<0.001
Lack of knowledge about migrant patients' countries and cultures	454 (34.7)	246 (33.3)	0.94 (0.78–1.14)	0.56	0.94 (0.77–1.15)	0.56
Lack of knowledge about medical and social services available for migrant patients	527 (40.2)	238 (32.8)	0.72 (0.60–0.87)	0.001	0.84 (0.69–1.03)	0.10
Lack of skills in communicating with patient from other languages and cultures	584 (44.5)	286 (39.1)	0.80 (0.67–0.96)	0.020	0.93 (0.76–1.12)	0.44
Patient's unrealistic expectations	392 (30.7)	230 (31.4)	1.03 (0.85–1.26)	0.76	0.94 (0.76–1.15)	0.52
Patient's lack of education	356 (27.5)	180 (24.7)	0.86 (0.70–1.06)	0.17	0.86 (0.69–1.07)	0.17
Lack of time	698 (53.1)	347 (47.7)	0.80 (0.67–0.96)	0.018	0.87 (0.72–1.05)	0.14
Bias or prejudice on the part of hospital staff	215 (16.5)	103 (14.1)	0.83 (0.64–1.07)	0.16	0.81 (0.62–1.05)	0.11
HUG not adapted to needs of migrant patients	199 (15.5)	90 (12.6)	0.79 (0.60–1.03)	0.083	0.81 (0.62–1.08)	0.15

*Adjusted by professional category, hospital department, function (senior doctor or nurse, vs. staff nurse or resident), and Swiss versus other citizenship.

## Discussion

After three years of activity aimed at improving staff knowledge and practices regarding care of migrant patients, not quite half of respondents had heard of the HUG Health for All Network in 2013. This is lower than we would have liked, but this may be due to the fact that our interdepartmental and interprofessional working group changed names half way through the project period. Only after the name change did we develop a logo and brochures. In hindsight, we probably should have formalized the group identity earlier in the project to facilitate its visibility.

Nonetheless, we observed several improvements in staff attitudes and knowledge, especially with regards to communicating across language barriers. We seem to have had little impact on respondents' comfort level with a number of other patient care tasks, including taking a social/cultural history of the patient, identifying and addressing potential sources of cultural misunderstanding, or negotiating a treatment plan that takes into consideration the patient's cultural beliefs, but this should perhaps not be surprising since our efforts focused on bringing attention to existing Migrant Friendly services at the HUG and better informing staff on how to use these services. Strengthening clinicians' patient care skills will require systematic integration of cultural competence topics into health professional curricula and innovative teaching approaches [34], [35], [36].

Time constraints and language barriers continue to be the most important sources of difficulty at our hospital. While effective use of interpreter services can contribute to better quality and more efficient communication with patients, organizing an appointment with an interpreter takes time. Even though respondents in 2013 preferred and used professional interpreters more often, use of patients' family members to translate and clinicians' use of their own foreign language skills did not diminish, probably reflecting the fact that these are the easiest and least costly solutions available. As Diamond [37] illustrated, even doctors in institutions with readily available interpreter services find it easier to “get by” without a professional interpreter, due to perceived time constraints. There is little our project can do to alleviate time constraints at the HUG, but facilitating access to professional interpreters, for example through telephone interpreting services, may help, and will be a focus of future efforts. However, telephone interpreting is not appropriate for all clinical situations. Face-to-face interpreters are generally recommended for complex, emotional or lengthy conversations, as well as for conversations involving more than 2 individuals. Concern has also been raised that the quality of communication may be compromised when the interpreter is over-the-phone rather than face-to-face. The phone interpreter lacks non-verbal cues that can contribute to more accurate interpreting, and physicians may tend to shorten their communication when the interpreter is over-the-phone [38].

The main weakness of our study is the poor response rate to the 2013 survey and the differences in respondent characteristics between the two surveys. We believe the main explanation for this is the imposed use of an internet survey sent to respondents' professional email addresses in 2013. As part of the paper-saving policy of the institution, all staff had recently been required to have an institutional email address, to which is sent most institutional communication, including salary statements. However, many staff do not have the time or opportunity to access their email during the workday (especially nurse aides), and others may not wish to access their work email at home. In addition, non-physician staff are rarely invited to participate in on-line survey research. In contrast, physicians use email often, and are frequently invited to participate in on-line surveys. It is likely that it took more motivation and a higher level of comfort with email and internet surveys to respond to the 2013 survey, and this appears to be reflected in the 2013 sample, which had proportionately more doctors and more respondents from the 4 departments with the highest number of migrant patients (Community Medicine, Psychiatry, Gyn/Obs and Adolescent and Child health).

We hope to have corrected these differences through statistical adjustment, but the possibility remains that the comparisons remain partially affected by unmeasured confounders. We are reasonably confident that unmeasured confounders would not explain the full extent of the differences that we observed, because the greatest differences were seen in areas where the program was most active, such as the promotion of interpreter services, and no differences were observed for domains that the program did not address. It is noteworthy that even though low participation in internet surveys is common [39], [40], this does not necessarily cause bias in the variables of scientific interest [41].

In addition, our pre/post survey design does not allow us to claim without a doubt that our project is responsible for the observed changes. It is possible that other factors influenced respondents' knowledge and practices, but we know of no other institutional-level activities aimed at migrant care issues that occurred during the project period. Finally, our results are limited to self-reported behavior. Had we had access to other sources of data, such as patient reports or service use statistics we might have obtained different results. However, such data were not available.

Our experience points to the importance of institutional commitment in ensuring equity and quality for diverse patient populations. A previous study at the HUG [42] showed that doctors and nurses often feel their efforts to provide quality care to patients are constrained and threatened by institutional factors outside their control, such as budget cuts, overwork and time constraints. Institutions must provide the resources, conditions and work environment necessary for clinicians to be able to identify and respond to the specific needs of migrant patients. Our program is innovative in that it uses a national-level project to gain leverage within the institution. The Federal Office of Public Health required co-funding from each of the participating hospitals, and a commitment to perennialize key project activities. The HUG directorate agreed to co-finance staff time, and provided us with invaluable support and technical input from the hospital communication service. We believe that this institutional commitment and financing, along with inter-departmental participation in all activities were important in creating and maintaining project visibility, and that such visibility contributes to developing a “migrant friendly” institutional culture. However, while projects such as ours may contribute to the provision of non-discriminatory, quality health care to all patients, it remains to be seen whether such efforts can be sustained in the face of shrinking budgets, increasing interpreting costs and growing anti-immigrant sentiment [43].

## Supporting Information

Questionnaire S1 English translation of the staff questionnaire.
(PDF)
