# Supplementary material for: A “Migrant Friendly Hospital” Initiative in Geneva, Switzerland: Evaluation of the Effects on Staff Knowledge and Practices
Source: PLoS One. 2014 Sep 8;9(9):e106758. doi: 10.1371/journal.pone.0106758 (PMC4157774; doi:10.1371/journal.pone.0106758)
Supplement: Questionnaire S1 — English translation of the staff questionnaire. (PDF) [file pone.0106758.s001.pdf]

Hôpitaux Universitaires de Genève

CARE OF MIGRANT/VULNERABLE PATIENTS
SPRING 2013

YOUR CLINICAL PRACTICE

Clinicians at the HUG may find it difficult to deal with the social and cultural diversity of patients. For each item below, please indicate whether it is a rare or common cause of difficulty for you in your professional practice.

If you work in pediatrics: items concerning patients relate to your experience working with children and families.

	Rare cause of difficulty ▼				Very frequent cause of difficulty ▼
1. Lack of experience with migrant patients	1	2	3	4	5
2. Lack of knowledge about migrant patients' countries and cultures	1	2	3	4	5
3. Lack of knowledge about medical and social resources for migrants in Geneva	1	2	3	4	5
4. Lack of skills in communicating with patients from other cultures and languages	1	2	3	4	5
5. The migrant patient's unrealistic expectations	1	2	3	4	5
6. The migrant patient's insufficient knowledge of French	1	2	3	4	5
7. The migrant patient's lack of knowledge about how the hospital functions	1	2	3	4	5
8. The migrant patient's low level of education	1	2	3	4	5
9. Lack of time to deal with language and cultural barriers	1	2	3	4	5
10. Bias or prejudice against migrant patients on the part of health care personnel at the HUG	1	2	3	4	5
11. The hospital's lack of adaptation to the needs and expectations of migrant patients	1	2	3	4	5
12. Lack of access to professional interpreters for communicating with migrant patients	1	2	3	4	5
13. Lack of patient brochures translated to migrant patients' languages	1	2	3	4	5

TRAINING

Have you ever received training on the following topics ?	Yes	No
14. Risks associated with the use of untrained interpreters	☐1	☐2
15. Procedures for organizing an appointment with a Geneva Red Cross interpreter	☐1	☐2
16. How to work with a community interpreter	☐1	☐2
17. Healthcare access for asylum seekers and undocumented migrants at the HUG	☐1	☐2
18. Social, medical and associative services for migrant patients in Geneva	☐1	☐2
19. Influence of the patient's religion on health care	☐1	☐2
20. History and culture of specific migrant populations	☐1	☐2

How interested would you be to receive training on the following topics:	Not at all interested				Very interested
21. Risks associated with the use of untrained interpreters	1	2	3	4	5
22. Procedures for organizing an appointment with a Geneva Red Cross interpreter	1	2	3	4	5
23. How to work with a community interpreter	1	2	3	4	5
24. Healthcare access for asylum seekers and undocumented migrants at the HUG	1	2	3	4	5
25. Social, medical and associative services for migrant patients in Geneva	1	2	3	4	5
26. Influence of the patient's religion on health care	1	2	3	4	5
27. History and culture of specific migrant populations	1	2	3	4	5

28. Would you like to receive training on any other topics regarding migrant care? Please specify:

____

____

YOUR OPINION

Please circle the number that best reflects your personal opinion.
Circle the number 1 if you are total agreement with the statement on the left, and circle the number 7 if you are in total agreement with statement on the right. If your opinion is somewhere in between, circle one of the numbers in the middle.

29. When migrants' customs and values differ from those of Switzerland :

Swiss institutions should adapt to the values and customs of migrants						Migrants should adapt to Swiss institutions
⮟						⮟
1	2	3	4	5	6	7

30. When the patient does not speak French :

The hospital should always make a professional interpreter available to the patient :						It's the patient's responsibility to find someone to translate
⮟						⮟
1	2	3	4	5	6	7

31. When the patient does not read French :

The hospital should provide written information in the patient's language						It is the patient's responsibility to understand written information provided by the hospital
⮟						⮟
1	2	3	4	5	6	7

RESOURCES FOR CARING FOR MIGRANT PATIENTS AT THE HUG

Questions 32 to 39

Listed below are several structures at the HUG aimed at migrant patients. For each one, please indicate if you have heard of this structure, and if you have previously had contact with it for a patient.

STRUCTURES	*Have you heard of it?*		*Have you had contact with it?*	
	OUI	NON	OUI	NON
32. **Mobile consultation for community care (CAMSCO), ex UMSCO**	☐1	☐2	☐1	☐2
33. Migrant Health Program/Center	☐1	☐2	☐1	☐2
34. Geneva Red Cross community interpreter service	☐1	☐2	☐1	☐2
35. Ethnopsychiatry consultation at the Department of Child and Adolescent Health	☐1	☐2	☐1	☐2
36. Migrant health consultation at the Department of Child and Adolescent Health	☐1	☐2	☐1	☐2
37. Cross-cultural consultation	☐1	☐2	☐1	☐2
38. Consultation for victims of torture and war	☐1	☐2	☐1	☐2
39. Clinical specialist nurse for migrant/vulnerable patients	☐1	☐2	☐1	☐2

40. Have you heard about the Health for All network at the HUG (previously referred to as the interdepartmental programme for migrant/vulnerable patients) ?

    Oui ☐1

    Non ☐2

41. Which of the following Health for All Network brochures have you seen?

Health for All Network brochures	*Have you seen it before?*	
	YES	NO
42. Brochure « Practical Information »	☐1	☐2
43. Brochure « Call a trained interpreter»	☐1	☐2

LANGUAGE BARRIERS

44. In your work at the HUG, how often do you see patients who speak little or no French?

Never ☐1
Rarely ☐2
Sometimes ☐3
Often ☐4

During the last 6 months, how often did you use each of the following to help you communicate with patients who did not speak French well?

	Never	1-10 times	11-20 times	More than 20 times
45. Patient's family members or friends (adults)	☐1	☐2	☐3	☐4
46. Children under 18 years of age	☐1	☐2	☐3	☐4
47. Bilingual hospital staff (with no interpreter training)	☐1	☐2	☐3	☐4
48. Myself, because I speak other languages than French	☐1	☐2	☐3	☐4
49. Geneva Red Cross community interpreter, face-to-face	☐1	☐2	☐3	☐4
50. Geneva Red Cross community interpreter, over the phone	☐1	☐2	☐3	☐4

51. If you have the choice, which of the following persons do you prefer to use to help communicate with patients when there is a language barrier?
Check only one :

☐1 Adult family or friends of the patient
☐2 Bilingual hospital staff
☐3 Geneva Red Cross community interpreters
☐4 I don't know

52. The service/department where you currently work (**Check only one**):

☐1 Encourages its staff to use Geneva Red Cross interpreters to communicate with patients who speak French poorly
☐2 Encourages its staff to look for *other* solutions for communicating with patients who speak French poorly (for example, ask a staff member or patient's family or friends to translate)
☐3 Gives no instructions on what to do when patients speak French poorly
☐4 I don't know

53. Have you heard of the national telephone interpreting service AOZ-Medios ?

Yes ☐1

No ☐2

54. If it was easily available at the HUG, how interested would you be in having access to a telephone interpreting service to communicate with your patients, available 24 hours and 7 days a week?

Not at all interested				Very interested
1	2	3	4	5

YOUR PROFESSIONAL PRACTICE

To what degree do you consider yourself competent to carry out the following tasks with a migrant patient (a foreign patient, born and bred in a country other than Switzerland)?

	Not at all competent				Very competent
55. Take a social history of the patient	1	2	3	4	5
56. Identify the patient's ability to read and write in French	1	2	3	4	5
57. Negotiate a treatment plan with the patient and his/her family	1	2	3	4	5
58. Evaluate the patient's understanding of his/her illness/health problem	1	2	3	4	5
59. Discuss the advantages and potential risks of complementary and traditional medicine practices with a patient who uses these	1	2	3	4	5
60. Identify the patient's cultural/religious habits that could have an impact on care	1	2	3	4	5
61. Work with a trained community interpreter	1	2	3	4	5
62. Explore the migrant patient's potentially traumatic experiences	1	2	3	4	5
63. Orient the migrant patient towards appropriate social and outpatient medical services	1	2	3	4	5

A FEW QUESTIONS ABOUT YOURSELF:

64. You work as (**only one response possible) :**

- ☐[1] Social worker or public health nurse (ISP)
- ☐[2] Head nurse (RS, ARS, RU)
- ☐[3] Nurse or nurse specialist
- ☐[4] Nurse’s aide or ASSC
- ☐[5] Resident physician
- ☐[6] Senior resident physician
- ☐[7] Attending physician/ head of service or department
- ☐[8] Psychologist
- ☐[9] Other health care professional (“PPS”): Group facilitator; art therapist; dietician; educator; ergotherapist; dental hygienist; music therapist; podiatrist; physiotherapist; psychomotor therapist; radiology technician)

65. In what department do you work? *Only one response possible: if you are affiliated with several departments, choose the department where you work the most or the one you most identify with)*

- ☐[1] Anesthesiology, Pharmacology and Intensive Care
- ☐[2] Surgery
- ☐[3] Child and Adolescent Health
- ☐[4] Gynecology and Obstetrics
- ☐[5] Imagery & Medical Information Sciences
- ☐[6] Community Medicine, Primary Care and Emergency Medicine
- ☐[7] Genetics and Laboratory
- ☐[8] Internal Medicine, Rehabilitation and Geriatrics
- ☐[9] Clinical Neurosciences
- ☐[10] Mental Health And Psychiatry
- ☐[11] Medical Specialties

66. How long have you worked at the HUG?

- ☐[1] Less than 1 year
- ☐[2] 1-5 years
- ☐[3] 6-10 years
- ☐[4] 11-20 years
- ☐[5] More than 20 years

67. You are:

- ☐[1] A man
- ☐[2] A woman

68. What is your nationality?

- ☐[1] Swiss only
- ☐[2] European only (EU + EFTA)
- ☐[3] Only non-European
- ☐[4] Double nationality: Swiss + European (EU + EFTA)
- ☐[5] Double nationality: Swiss and non-European
- ☐[6] Double nationality: European + European
- ☐[7] Double nationality: European + non-European
- ☐[8] Double nationality: non-European + non-European

69. What language do you speak/understand the best? Choose only one, even if you speak several languages.

☐[1] French	☐[10] Farsi (Persian)	☐[19] Romanian
☐[2] English	☐[11] Hindi	☐[20] Russian
☐[3] Albanian	☐[12] Italian	☐[21] Somali
☐[4] German	☐[13] Kurdish	☐[22] Swiss German
☐[5] Amharic	☐[14] Lingala	☐[23] Tamil
☐[6] Arab	☐[15] Urdu	☐[24] Tibetan
☐[7] Bosnian/Serbian/Croatian	☐[16] Portuguese	☐[25] Tigrigna
☐[8] Chinese (Mandarin)	☐[17] Romani	☐[26] Turkish
☐[9] Spanish	☐[18] Romansh	☐[27] OTHER __________

Thank you for your collaboration!

Please return the questionnaire in the pre-addressed, stamped envelope enclosed.

For more information, please contact:

Dr. Sophie Durieux-Paillard

Migrant Health Program, Primary Care Service, HUG

Sophie.Durieux@hcuge.ch

Tél : 022 382 33 42
